# The Vitamin B1 Metabolism of *Staphylococcus aureus* Is Controlled at Enzymatic and Transcriptional Levels

**DOI:** 10.1371/journal.pone.0007656

**Published:** 2009-11-03

**Authors:** Ingrid B. Müller, Bärbel Bergmann, Matthew R. Groves, Isabel Couto, Leonard Amaral, Tadhg P. Begley, Rolf D. Walter, Carsten Wrenger

**Affiliations:** 1 Department of Biochemistry, Bernhard Nocht Institute for Tropical Medicine, Hamburg, Germany; 2 European Molecular Biology Laboratory-Hamburg Outstation, Hamburg, Germany; 3 Unit of Mycobacteriology, Instituto de Higiene e Medicina Tropical, Universidade Nova de Lisboa (IHMT/UNL), Lisboa, Portugal; 4 Department of Chemistry, Texas A&M University, College Station, Texas, United States of America; National Institute of Allergy and Infectious Diseases, National Institutes of Health, United States of America

## Abstract

Vitamin B1 is in its active form thiamine pyrophosphate (TPP), an essential cofactor for several key enzymes in the carbohydrate metabolism. Mammals must salvage this crucial nutrient from their diet in order to complement the deficiency of *de novo* synthesis. In the human pathogenic bacterium *Staphylococcus aureus,* two operons were identified which are involved in vitamin B1 metabolism. The first operon encodes for the thiaminase type II (TenA), 4-amino-5-hydroxymethyl-2-methylpyrimidine kinase (ThiD), 5-(2-hydroxyethyl)-4-methylthiazole kinase (ThiM) and thiamine phosphate synthase (ThiE). The second operon encodes a phosphatase, an epimerase and the thiamine pyrophosphokinase (TPK). The open reading frames of the individual operons were cloned, their corresponding proteins were recombinantly expressed and biochemically analysed. The kinetic properties of the enzymes as well as the binding of TPP to the *in vitro* transcribed RNA of the proposed operons suggest that the vitamin B1 homeostasis in *S. aureus* is strongly regulated at transcriptional as well as enzymatic levels.

## Introduction

The pathogenic bacterium, *Staphylococcus aureus*, is responsible for a wide spectrum of human and animal diseases, ranging from benign skin infections to severe diseases, such as arthritis, osteomyelitis, endocarditis and fatal sepsis [1]. The infections are difficult to treat and often relapse even after prolonged and adapted antibiotic therapy, suggesting that *S. aureus* has developed specific strategies for persistence [2], [3]. Although not traditionally considered as an intracellular pathogen, the bacterium can survive in a variety of cells, hiding from the human immune system [4].

Vitamin B1, or thiamine, is present in all organisms as an essential cofactor of several key enzymes such as pyruvate dehydrogenase, 2-oxoglutarate dehydrogenase, branched-chained-2-oxoacid dehydrogenase and transketolase [5]. Thiamine pyrophosphate (TPP), the active form of the cofactor, must be either salvaged or synthesised *de novo*. Humans and other mammals depend completely upon the uptake of vitamin B1 from their diet, and the deficiency of this essential nutrient results in Wernicke's disease and beriberi. Plants, bacteria and the protozoan parasite *Plasmodium* synthesize vitamin B1 *de novo* via two pathways, which are merged to thiamine monophosphate (TMP) by thiamine phosphate synthase (ThiE) [6], [7], [8]. Whereas in *E. coli* TMP is subsequently phosphorylated to TPP by the thiamine phosphate kinase (ThiL) [6], yeast and *Plasmodium* do not possess ThiL, but instead a thiamine pyrophosphokinase (TPK). Previous data from *P. falciparum* also demonstrate that TMP synthesised *de novo* is dephosphorylated prior to pyrophosphorylation by TPK [9].

Here we report the identification and characterisation of two operons encoding enzymes involved in the biosynthesis and degradation of vitamin B1 in *S. aureus*, including a GTPase, dephosphorylating TMP.

## Results and Discussion

### Identification of Genes Involved in Vitamin B1 Metabolism

Extensive BLAST searches within the *S. aureus* genome database (http://www.sanger.ac.uk/Projects/S_aureus/) using the respective homologous derived enzymes from other organisms identified the open reading frames (ORFs) of 4-amino-5-hydroxymethyl-2-methylpyrimidine (HMP) kinase (*Sa*ThiD), 5-(2-hydroxyethyl)-4-methylthiazole (THZ) kinase (*Sa*ThiM), thiaminase (*Sa*TenA), thiamine monophosphate (TMP) synthase (*Sa*ThiE), thiamine pyrophosphate (TPP) kinase (*Sa*TPK). Furthermore, sequence analyses within the *S. aureus* genome revealed that all deduced proteins appear as single copy genes.

The ORFs of *thiD*, *thiM*, *thiE*, and *tenA* consist of 831, 729, 642 and 690 bp resulting in corresponding proteins of 276, 263, 213 and 229 amino acid residues with calculated molecular masses of 30.2, 28, 23.4 and 26.8 kDa. Interestingly, these four ORFs are found in close proximity to each other, only separated by 2–5 bp, suggesting an operon-like organisation. In bacteria TenA is almost always found in a cluster with ThiD or – as reported from *Saccharomyces cerevisiae* – both enzymes are fused and form a bifunctional protein [10]. The bifunctional *S. cerevisiae* Thi6-p gene encodes the ThiE domain at the N-terminus and the ThiM domain at the C-terminus [11].

**Figure 1:**
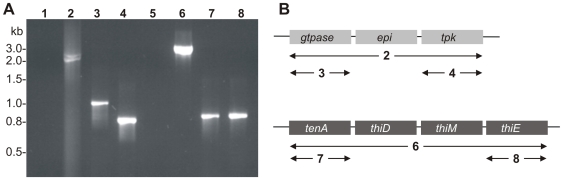
Reverse transcriptase (RT-) PCR on total *S. aureus* RNA. RT-PCR was carried out to evaluate whether the open reading frames of the *S. aureus* vitamin B1 metabolism are clustered and thereby suggested to be organized in operons. (A) Amplification of the cluster consisting of the ORFs *gtpase*, *epi* and *tpk* using the primers *Sa*GTPase-IBA3-S and *Sa*TPK-IBA3-AS (lane 2); as controls, the PCR was either carried out without RT step (lane 1) or amplification of the flanking ORFs was performed with the primers *Sa*GTPase-IBA3-S and *Sa*GTPase-IBA3-AS (lane 3) and *Sa*TPK-IBA3-S and *Sa*TPK-IBA3-AS (lane 4). The ORFs of the operon consisting of *tenA*, *thiM*, *thiD* and *thiE* were amplified using the primers *Sa*TenA-IBA3-S and *Sa*ThiE-IBA3-AS (lane 6). As controls the PCR was carried out without RT step (lane 5) and amplification of the flanking open reading frames was performed either with the primer *Sa*TenA-IBA3-S and *Sa*TenA-IBA3-AS (lane 7) or the primer *Sa*ThiE-IBA3-S and *Sa*ThiE-IBA3-AS (lane 8). (B) The organisation of the proposed operons is schematically illustrated. Note: Numbering of figure 1B refers to the respective lane number of figure 1A.

In *Bacillus subtilis* TenA is part of the operon that encodes the genes for THZ biosynthesis [12], [13]. In order to analyse the organisation in *S. aureus* reverse transcriptase PCR was carried out on total RNA, using primers flanking the respective ORFs (Fig. 1A/B). The PCR products obtained clearly emphasised the presence of *tenA*, *thiM*, *thiD* and *thiE* within a cluster.

In contrast, the *tpk* gene was not found within this cluster, but was identified in a different cluster consisting of two further ORFs in close proximity to *tpk*; one with homology to bacterial ribulose 5-phosphate epimerases and the other to ribosome-associated GTPases (Fig. 1B). The *gtpase* and *tpk* genes are separated by 663 bp, of which 645 bp correspond to a predicted ribulose 5-phosphate epimerase (*epi*). The ORFs of *gtpase* and *tpk* consist of 876 and 642 bp, resulting in proteins of 291 and 213 amino acid residues with calculated molecular masses of 33.8 and 23.9 kDa, respectively (Fig. 1A/B). RT-PCR was performed to examine the organisation of these ORFs, and proposed the occurrence as a clustered gene organisation (*gtpase* – *epi* – *tpk*; Fig. 1B).

In *E. coli* thiamine is phosphorylated in two consecutive steps by thiamine kinase (YcfN) and thiamine phosphate kinase (ThiL) [14], [15]. In eukaryotes (such as yeast and the malaria pathogen *P. falciparum*) thiamine is diphosphorylated by TPK [9], [10], [16]. The presence of a TPK in *S. aureus* suggested a similar vitamin B1 metabolism, as is known for yeast. Extensive BLAST searches did not identify any homology to ThiL in the *S. aureus* genome database.

### Biochemical Analysis of the *S. aureus* Proteins

**Figure 2:**
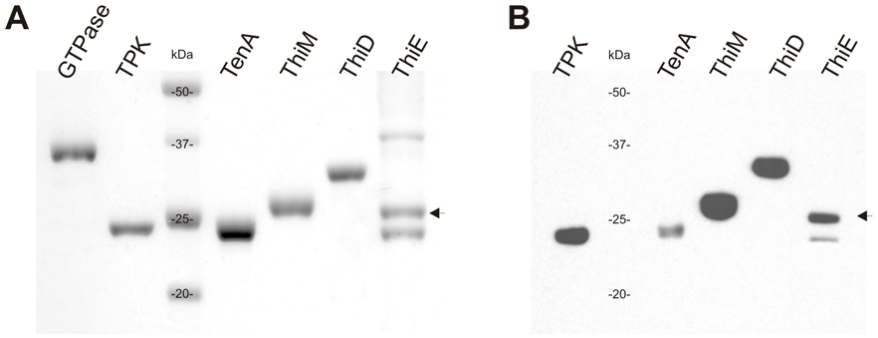
SDS-PAGE- and western blot-analysis of the recombinant enzymes of the *S. aureus* vitamin B1 metabolism. (A) SDS-PAGE of the affinity chromatography purified recombinant enzymes and (B) corresponding western blot analysis using a monoclonal anti-Strep antibody at a dilution of 1∶5,000 according to material and methods. GTPase; TPK, thiamine pyrophosphokinase; TenA, thiaminase II; ThiM, THZ kinase; ThiD, HMP kinase; ThiE, TMP synthase. The arrow indicates the *Sa*ThiE protein.

**Figure 3:**
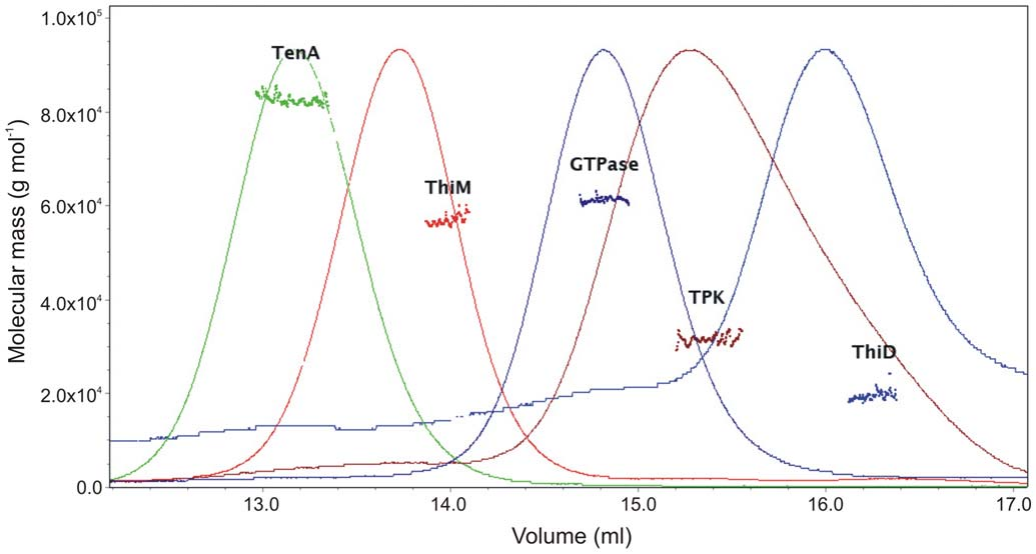
Static light scattering of the recombinant proteins. Size exclusion chromatography was performed with GTPase (purple); TPK (brown); TenA (green); ThiM (red); and ThiD (blue) and the masses measured by static light scattering. Molecular masses are given on the left.

The respective ORFs were cloned and recombinantly expressed in *E. coli* as soluble C-terminal Strep-Tag fusion proteins. The expressed polypeptides were purified by affinity chromatography and the purity of the recombinant enzymes was assessed by SDS-PAGE and western blotting - using a monoclonal anti-Strep antibody (Fig. 2A/B). To elucidate the oligomeric state, the proteins were applied to gel filtration on a Superdex S-200 column followed by static light scattering (SLS). Proteins eluted as single peaks and their corresponding molecular mass, as well as oligomeric state, are shown in Table 1 and Figure 3. Similar to homologues from other organisms *Sa*ThiD is a dimer and *Sa*TenA a trimer, *in vitro* [7], [17], [18]. Interestingly *Sa*ThiM was shown to be dimeric *in vitro*, whereas the *B. subtilis* and plasmodial counterparts are trimeric or monomeric, respectively [7], [19]. Both the *Sa*GTPase and the *Sa*TPK eluted as monomers. The respective plasmodial enzymes are dimers, which has also been reported for the mammalian and yeast TPK. However, phosphatases are found in various oligomeric states [16], [20], [21], [22].

**Table 1:** Kinetic properties of the *S. aureus* enzymes.

Parameter	*Sa*THiD	*Sa*ThiM	*Sa*THiE	*Sa*TenA	*Sa*TPK
Specific activity for	HMP	THZ	ND	Thiamine	Thiamine
(nmol min^−1^ mg^−1^)	23.5±1.4	4883±488		4.6±0.9	22.5±3.6
Substrate acceptance	HMP(-P), ATP, Bacimethrin	THZ, ATP	HMP-PP, THZ-P	Thiamine	Thiamine, ATP
*K*_m_^app^-value for	HMP	THZ	HMP-PP	Thiamine	Thiamine
(µM)	635±20	44±5	145±36	256±44	394±74
*k*_cat_-value (min^−1^)	0.7±0.05	137±13	ND	0.1±0.02	0.5±0.07
Calculated molecular mass - SLS analysis (kDa)	61.2±7.9	56.7±8.5	ND	82.9±9.9	29.8±3.8
Proposed oligomeric state	Dimer	Dimer	ND	Trimer	Monomer

The kinetic parameters were determined as described in the material and methods section. The results are the means of at least four independent experiments given with standard derivation (SD). Note: Due to limiting expression yield SLS analysis of ThiE was not performed. ND, not determined.

The biochemical properties of the *S. aureus* proteins were characterised by determining specific activities, apparent *K*_m_-values as well as substrate profiles. The specific activity as well as the *K*_m_-value for HMP of *Sa*ThiD is within the range for those reported for the *E. coli* and *B. subtilis* counterparts [23], [24] (Table 1). It has been reported that ThiD is able to phosphorylate HMP to HMP-P and in a second step HMP-P to HMP-PP [7], [17]. Interestingly, when HMP-P was employed as substrate for the *S. aureus* enzyme, only trace amounts of HMP-PP synthesis was detected - suggesting similarity to the ThiD proteins of *E. coli* and *P. falciparum*. Both proteins showed clear preferences for HMP and the second reaction step leading to HMP-PP was clearly reduced in *P. falciparum* by a factor of up to 80 times [7]. Additionally, the antibiotic bacimethrin, previously reported as HMP analogue and substrate for ThiD from other organisms [7], [25], was tested on *Sa*ThiD. Indeed, bacimethrin acts as a substrate for *Sa*ThiD revealing a specific activity of 14.6±1.2 nmol min^−1^ mg^−1^ protein, which is within the same range as the native substrate HMP (Table 1). Channelled into the vitamin B1 biosynthesis of *S. aureus,* the pyrophosphorylated bacimethrin exchanges HMP-PP to give a non-functional TMP derivative, that may subsequently interfere with the function of TPP-dependent enzymes [7], [25], [26]. A similar strategy was described for targeting vitamin B6 dependent enzymes in *P. falciparum* [27].

**Figure 4:**
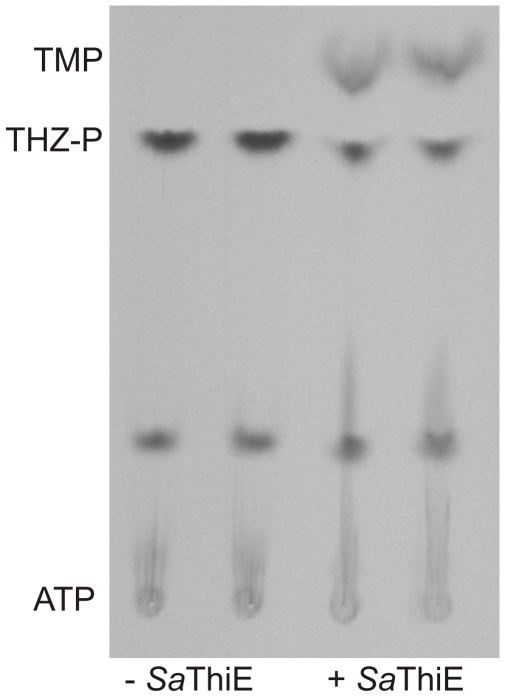
Thin layer chromatography of the *Sa*ThiE reaction product. The *Sa*ThiE reaction was carried out using HMP-PP and THZ-[^33^P] as substrates and the reaction products were analysed by their respective R_f_-values of 0.25 for TMP and 0.32 for THZ-P. As control the reaction was performed without addition of *Sa*ThiE. Note: The ATP spots were the substrates of the ThiM catalysis to give THZ-[^33^P] as described in the material and methods section.

**Figure 5:**
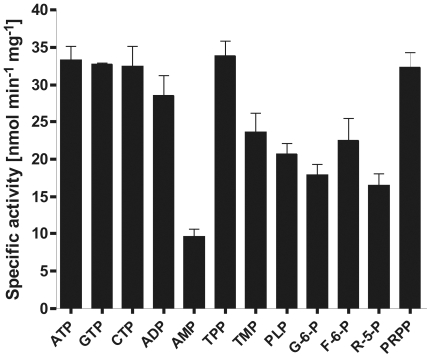
Substrate profile of the *S. aureus* GTPase. Standard assay conditions were used as described in material and methods. The results are the means of at least four independent experiments and the standard derivations are given. Adenosine triphosphate, ATP; Guanine triphosphate, GTP; cytidine triphosphate, CTP; Adenosine diphosphate, ADP; Adenosine monophosphate, AMP; thiamine pyrophosphate, TPP; thiamine monophosphate, TMP; pyridoxal 5-phosphate, PLP; glucose 6-phosphate, G-6-P; fructose 6-phosphate, F-6-P; ribose 5-phosphate, R-5-P; phosphoryl-ribose pyrophosphate, PRPP.

*Sa*ThiM showed strict substrate preferences for THZ and ATP and does not accept HMP as substrate. Its apparent *K*_m_-value for THZ is within the range for the homologues in *B. subtilis* and *P. falciparum*, 34 µM and 68 µM, respectively (Table 1), whereas the specific activity of the *S. aureus* enzyme is about 18 times higher when compared to that of the plasmodial enzyme [7], [19], [28]. Recombinant expression of ThiE is limited as known from the *E. coli* and the plasmodial counterpart. To date, only ThiE of *B. subtilis* has been recombinantly expressed at sufficient levels [7], [29]. Due to the presence of three different bands in SDS-PAGE analysis (Fig. 2A), western blotting was performed and identified a hybridisation signal of 25 kDa, which is in good agreement with the predicted molecular mass of *Sa*ThiE (including the Strep-tag) and a probable slightly smaller breakdown product of *Sa*ThiE (Fig. 2B). *Sa*ThiE revealed substrate stringency for HMP-PP and no activity was observed in the presence of either HMP or HMP-P. The *K*_m_-value for HMP-PP was calculated to be 145 µM (Table 1). As clearly shown in Figure 4, the ThiE reaction generates TMP, but TMP is not the active form of the cofactor and has to be further phosphorylated to TPP. TPP is synthesised either by ThiL, which directly phosphorylates TMP [6], or alternatively by the TPK after dephosphorylation of TMP. Since no ORF encoding for ThiL was found in the genome database of *S. aureus*, it is suggested that TMP has to be dephosphorylated prior to pyrophosphorylation by TPK, as reported in the vitamin B1 metabolism of *P. falciparum* [30]. So far no specific TMP phosphatase has been reported. However, an ORF with homology to ribosome associated GTPases was identified and biochemically characterised on the transcript on which *Satpk* is found. The obtained data clearly show that the *S. aureus* GTPase is not restricted to GTP and exhibits a broad substrate spectrum. Beside GTP and other tri- and diphosphorylated nucleotides, further small molecules such as thiamine pyrophosphate and phosphoryl-ribose pyrophosphate are favoured. Thiamine monophosphate, pyridoxal 5-phosphate, AMP and the sugars glucose 6-phosphate, ribose 5-phosphate and fructose 6-phosphate are also substrates (Fig. 5). The fact that *Sagtpase* is separated from *Satpk* on the operon by an additional ORF, encoding for a putative ribulose 5-phosphate epimerase, suggests that *Sa*GTPase might also participate in other processes, which awaits further analysis. However, the fact that *Sagtpase* and *Satpk* are encoded on the same transcript and the GTPase accepts TMP as substrate (Fig. 5) emphasises an involvement of this enzyme in vitamin B1 metabolism.

After dephosphorylation, thiamine is diphosphorylated by thiamine pyrophosphokinase to TPP, the active form of vitamin B1. *Sa*TPK accepts only thiamine as substrate and reveals a specific activity of 22 nmol min^−1^ mg^−1^ protein, which is in the same range as the plasmodial counterpart, but approximately one-quarter of the mouse TPK [30], [31] (Table 1). The phosphorylated form, TMP, is not a substrate of the *Sa*TPK. Interestingly the *K*_m_-value calculated for the *S. aureus* enzyme is about 5-fold higher when compared to its plasmodial counterpart. Furthermore, the *K*_m_-value for thiamine of the *B. subtilis* and mammalian enzymes, which were determined to be 20 µM and 6 µM, respectively, are approximately 20- and 65-times lower than the value of the *S. aureus* TPK [14], [32].

So far two thiaminases have been reported, thiaminase I and thiaminase II [15]. Thiaminase I degrades thiamine in the presence of organic nucleophiles such as aniline, quinoline or pyridine into THZ and a HMP-nucleophile adduct [33], while thiaminase II is able to split thiamine into THZ and HMP in the presence of water [18]. Recently, thiaminase II was shown to be involved in pyrimidine salvage from degraded thiamine [34]. Results presented here suggest that the *S. aureus* TenA belongs to the second type of thiaminases and indeed analysis of its biochemical properties showed independence from specific nucleophiles. *Sa*TenA accepts thiamine as substrate and not its phosphorylated forms TMP and TPP. The specific activity was determined to be about 5 nmol min^−1^ mg^−1^ protein and the *K*_m_-value for thiamine was calculated to be 250 µM (Table 1). Both *Sa*TenA and *Sa*TPK compete for thiamine as a substrate. The *K*_m_-values of *Sa*TenA and *Sa*TPK for thiamine are in a comparable range *in vitro*, however the specific activity of the TPK is about 5-fold higher than that of TenA (Table 1). Hence, one could speculate that synthesis of TPP might be preferred in *S. aureus*. Another issue is the import of extra-cellular thiamine. Although *S. aureus* strains depending on external thiamine supply have been observed [35], there are no reports of thiamine uptake capabilities to date, which would – in additional to *de novo* synthesis - feed the intracellular thiamine pool in *S. aureus*. Interestingly, *S. aureus* is known to be adhesive to erythrocytes - a source of thiamine [36], [37] - which might emphasise uptake of this nutrient from red blood cells.

**Figure 6:**
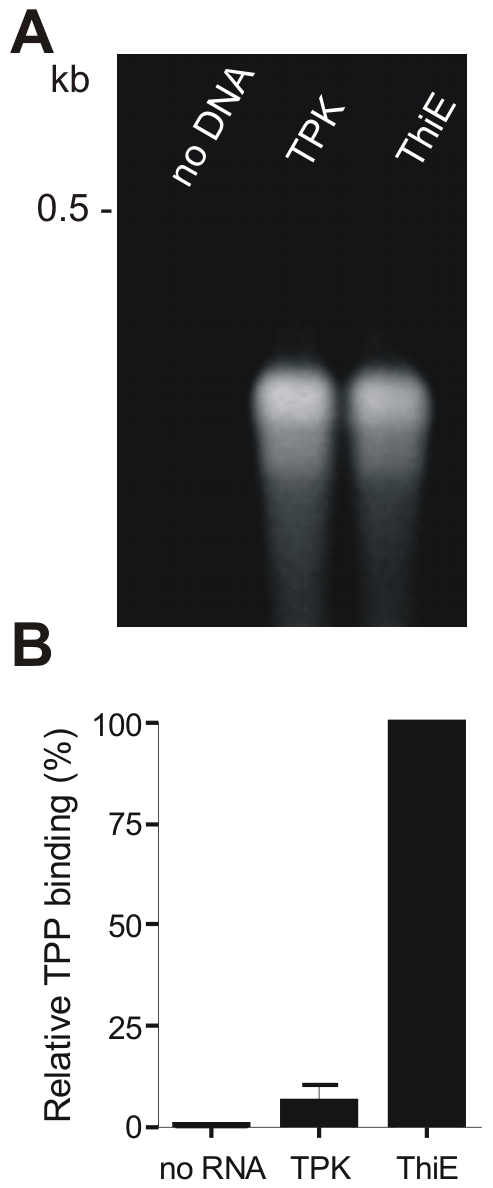
Analysis of TPP binding to its proposed THI boxes. (A) *In vitro* transcription of the potential 5′UTR −225 to −21 (relative to the translation start-site) of the operons consisting of *gtpase*, *epi* and *tpk* (TPK) and *tenA*, *thiM*, *thiD* and *thiE* (ThiE) using the MEGAscript *in vitro* transcription kit (Ambion, USA). As control the *in vitro* transcription was performed without DNA template (no DNA). (B) Relative binding affinity of TPP to the *in vitro* transcripted RNAs. The respective RNAs were denaturated, refolded and incubated with TPP [38]. Subsequently unbound TPP was separated from bound TPP by filtration and the TPP was oxidised to thiochrome. The fluorescence of thiochrome was detected at an excitation wavelength of 365 nm and at an emission wavelength of 455 nm. Data shown are from at least three independent experiments and expressed as percentage of the 5′UTR RNA of the *tenA*, *thiM*, *thiD* and *thiE* operon. No RNA, without RNA (control); TPK, 5′UTR sequence of the *gtpase*-*epi*-*tpk* operon; ThiE, 5′UTR sequence of the *tenA*-*thiM*-*thiD*-*thiE* operon.

It has been reported, that bacterial thiamine biosynthesis proteins are regulated at the transcriptional level by the binding of TPP to the riboswitch (THI-Box) on their respective mRNA, located mainly within the 5′ untranslated regions (UTR) [38], [39], [40]. Sequence alignments of the proposed *gtpase*-*epi*-*tpk* operon as well as the *tenA*-*thiM*-*thiD*-*thiE* operon identified homologies to the *E. coli* THI-Box. In order to verify the occurrence of THI-Boxes *in vitro* transcription of the potential 5′UTR sequences was performed and analysed for TPP binding according to [38] (Fig. 6A). The RNA obtained was subsequently incubated with an equimolar amount of TPP. Bound and unbound TPP were separated by filtration and the ratio determined by measuring the fluorescence of oxidised TPP (thiochrome) [38], [41]. The relative binding capacity of TPP to the 5′UTR of *tenA*-*thiM*-*thiD*-*thiE* sequence was about 28% of the total amount of applied TPP, which is approx. 12% less than that of the *E. coli thiM* leader RNA [38]. As shown in Figure 6B binding of TPP to the *tenA*-*thiM*-*thiD*-*thiE* RNA leader sequence (expressed as 100% TPP binding) is about 15-times elevated in comparison to the proposed 5′UTR sequence of the *gtpase*-*epi*-*tpk* operon, suggesting that transcriptional regulation via a THI-Box is likely to occur for the *S. aureus tenA*-*thiM*-*thiD*-*thiE* operon. Since TPP binding to the 5′UTR sequence of the *gtpase*-*epi*-*tpk* cluster is rather limited, regulation of the vitamin B1 homeostasis in *S. aureus* might not completely rely on transcriptional control via a riboswitch.

**Figure 7:**
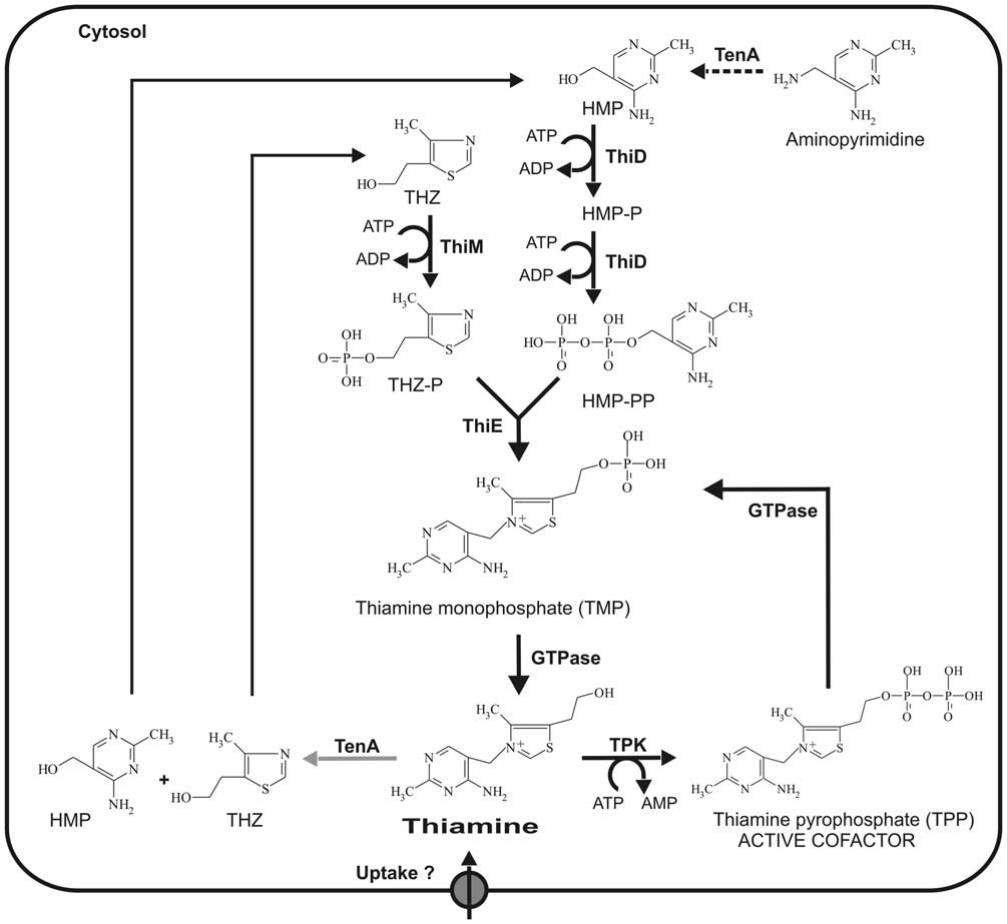
Schematic illustration of the proposed vitamin B1 metabolism in *S. aureus*. TenA is suggested to be involved in pyrimidine salvage leading to HMP as indicated by a dotted arrow [34]. However, TenA is also capable to degrade thiamine into HMP and THZ in *S. aureus* (grey arrow).

As deduced from biochemical analysis of *Sa*GTPase (located on the *gtpase*-*epi*-*tpk* operon), the enzyme not only dephosphorylates TPP to produce thiamine, which can be further degraded by TenA (located on the *tenA*-*thiM*-*thiD*-*thiE* operon) (Fig. 1 and 7), but accepts also *de novo* synthesised TMP, which is generated by enzymes again encoded on the *tenA*-*thiM*-*thiD*-*thiE* operon. Moreover, at an enzymatic level the biosynthetic enzyme TPK competes with the catabolic enzyme TenA for the same substrate thiamine (Fig. 7). Thus, regulation of the vitamin B1 homeostasis is probably controlled at enzymatic - as indicated above - and transcriptional levels, which is emphasised by the proposed THI-Box of the *tenA*-*thiM*-*thiD*-*thiE* operon. However, the precise mechanism of the regulation of these operons requires further experiments employing for example reverse genetics to analyse null-mutants.

## Materials and Methods

### Materials

Restriction enzymes and ligase were purchased from New England Biolabs, USA. Oligonucleotides were obtained from Operon, Germany. The cloning vector pASK-IBA3, Strep-Tactin-Sepharose, anhydrotetracycline (AHT) and desthiobiotin were from IBA (Institut für Bioanalytik, Germany). [γ-^33^P]-ATP (3000 Ci mmol^−1^) and [8-^14^C]-ATP (50 Ci mmol^−1^) were from Hartmann Biosciences, Germany. PEI cellulose F Polygram sheets were purchased from MERCK, Germany. HMP, HMP-P and HMP-PP were synthesised according to [42]. All other chemicals used were from Sigma, Germany.

### Cloning of the SaThiD, SaThiM, SaThiE, SaTenA, SaGTPase and SaTPK

The open reading frames (ORFs) encoding for ThiD, ThiM, TenA, TPK, GTPase and ThiE were amplified by PCR from *S. aureus* ATCC25923 genomic DNA using the sequence specific antisense and sense oligonucleotides as indicated in Table 2. The PCRs for the constructs were performed by using *Pfu*-Polymerase (Invitrogen, Germany) and the following PCR-program: Denaturation for 5 min at 95°C, followed by 35 cycles of 45 s 95°C, 30 s at 52°C, 2–3 min at 68°C. The generated PCR products were digested with *Bsa*I and cloned into the *E. coli* expression plasmid pASK-IBA3 previously digested with the same enzyme, resulting in the expression constructs *Sa*ThiD-IBA3, *Sa*ThiM-IBA3, *Sa*ThiE-IBA3, *Sa*TenA-IBA3, *Sa*GTPase-IBA3 *and Sa*TPK-IBA3. The plasmid pASK-IBA3 encodes for a C-terminal Strep-Tag that allows one-step purification of the recombinant fusion proteins using Strep-Tactin-Sepharose [43]. The nucleotide sequences of all clones were verified by automated nucleotide sequencing (MWG, Germany). Nucleotide and amino acid analyses were performed with the help of Generunner. In order to evaluate genetic organisation of the genes involved in *S. aureus* vitamin B1 metabolism reverse transcriptase PCR (RT-PCR) was carried out on total RNA using the Superscript III One-Step RT-PCR-System according to the manufacturer's recommendation (Invitrogen, Germany) and the RT-PCR-program: 30 min at 45°C, 2 min at 95°C followed by 35 cycles of 15 s 95°C, 30 s at 52°C, 3 min at 68°C.

**Table 2:** Oligonucleotides used for RT-PCR and for amplification of the potential 5′UTRs as well as for cloning of the ORFs *Sa*ThiD, *Sa*ThiM, *Sa*ThiE, *Sa*TenA, *Sa*GTPase and *Sa*TPK.

Name	Oligonucleotide sequence (5′→ 3′)
SaThiD-IBA3-S	GCGCGCGGTCTCGAATGATTAAACCTAAAATAGCATTAACC
SaThiD-IBA3-AS	GCGCGCGGTCTCGGCGCTTTTAGATAATTCATCGTCTAATCC
SaThiM-IBA3-S	GCGCGCGGTCTCGAATGAATTATCTAAATAACATACGTATTG
SaThiM-IBA3-AS	GCGCGCGGTCTCGGCGCTTTCCACCTCTTGAATGCGAATCCG
SaThiE-IBA3-S	GCGCGCGGTCTCGAATGTTTAACCAATCGTATCTAAATGTG
SaThiE-IBA3-AS	GCGCGCGGTCTCGGCGCTATTATTAAAAAAATCTTTGAATCG
SaTenA-IBA3-S	GCGCGCGGTCTCGAATGGAATTTTCACAAAAATTGTACC
SaTenA-IBA3-AS	GCGCGCGGTCTCGGCGCTATCATTTACTTTTCCTCCAAATTCC
SaGTPase-IBA3-S	GCGCGCGGTCTCGAATGAAGACAGGTCGAATAGTG
SaGTPase-IBA3-AS	GCGCGCGGTCTCGGCGCTATATCTAACCTTTCTATTTG
SaTPK-IBA3-S	GCGCGCGGTCTCGAATGCATATAAATTTATTATGTTCTGATCG
SaTPK-IBA3-AS	GCGCGCGGTCTCGGCGCTATTTAAATCTGTACTTCTAATTTGC
SaThiEoper+T7-S	TAATACGACTCACTATAG ATGTTGAAACCGTTTGCTAAGATTAATTT
SaThiEoper-T7-AS	AAAAAAACTACTTCCAACATGAAAGTAGTTTG
SaTPKoper+T7-S	TAATACGACTCACTATAG ATAATTGAAGTAAATGTACCGAGGTTTC
SaTPKoper-T7-AS	TTACTGCATTTATTATATCAAAGACTGG

Restriction sites or T7 promotor consensus sequences are underlined, respectively.

### Expression and Purification of the SaThiD, SaThiM, SaThiE, SaTenA, SaGTPase and SaTPK

The expression cells *E. coli* BLR (DE3) (Stratagene, Germany) were transformed with the cloned *S. aureus* ThiD, ThiM ThiE, TenA, GTPase and TPK constructs. Single colonies were picked and grown overnight in Luria-Bertani medium. The bacterial culture was diluted 1∶100 and grown at 37°C until the A_600_ reached 0.5. The expression was initiated with 200 ng ml^−1^ of anhydrotetracycline and the cells were grown for 4 hours at 37°C before being harvested. The cell pellet was re-suspended in 100 mM Tris-HCl, pH 8.0, 100 mM NaCl, sonicated, and centrifuged at 50,000 x g for 1 hour at 4°C. The recombinant Strep-Tag fusion protein was purified according to the manufacturer's recommendation (Institut für Bioanalytik, Germany). The eluted proteins from the affinity chromatography were analyzed by SDS-PAGE, and the protein was visualised by Coomassie staining [44]. The concentration of the purified recombinant protein was determined according to Bradford [45]. Western blot analysis was performed by loading the purified recombinant proteins of *S. aureus* onto 12.5% SDS-PAGE and subsequent transfer onto nitrocellulose membranes (Schleicher and Schüll, Germany). Briefly, western blots were incubated with the monoclonal anti-Strep antibody (IBA, Germany) at dilution of 1∶5,000. As secondary antibody, anti-mouse horseradish peroxidase-labelled goat antibody (Invitrogen, Germany) was used at a dilution of 1∶20,000. The hybridisation signals were visualised on X-ray films (Retina, Germany) using the ECL plus detection system according to the manufacturer's instructions (Amersham Biosciences, Germany).

### Molecular Mass of *S. aureus* ThiD, ThiM, TenA, GTPase and TPK

The molecular mass and oligomeric state of *SaThiD, SaThiM, SaTenA, SaGTPase and SaTPK* were assessed by analysing the affinity purified proteins using static light scattering. A miniDAWN Tristar (Wyatt Technologies, USA), was connected immediately downstream of a Superdex 200 10/30 size exclusion column (GE Healthcare) previously equilibrated with 100 mM Tris-HCl buffer, pH 8 containing 150 mM NaCl [46]. The collected SLS data were analysed using the manufacturer's recommended software (ASTRA V), based upon absorption coefficients calculated from the linear sequence of the recombinant proteins according to [47].

### Enzyme Assays for ThiD, ThiM, TenA, GTPase, ThiE and TPK

Analysis of *Sa*ThiD was carried out in a standard assay consisting of 100 mM potassium phosphate buffer, pH 7.5, 2 mM MgCl_2_, and 1 mM [75 nCi γ-^33^P]-ATP and 1 mM 4-amino-5-hydroxymethyl-2-methylpyrimidine (HMP) in a volume of 100 µl [7]. For analysis of the substrate specificity, the standard assay was conducted at 400 µM HMP or HMP-P. Kinetic studies of *Sa*ThiD were performed under standard assay conditions at varying concentrations of HMP between 0–2 mM at 1 mM [γ-^33^P]-ATP and subsequently the reaction products were separated by thin layer chromatography as described for the ThiM assay.

Kinetic analysis of *Sa*ThiM was carried out in 100 mM potassium phosphate buffer, pH 7.5, 1 mM MgCl_2_, 0.5 mM ATP and 400 µM 5-(2-hydroxyethyl)-4-methylthiazole (THZ). Kinetic studies were performed by varying concentrations of THZ between 0–600 µM at 500 µM [γ-^33^P]-ATP or [8-^14^C]-ATP [7]. The reaction mixture was incubated at 37°C for 0 to 30 min, stopped by heating at 95°C for 2 min. 10 µl aliquots of the supernatants were spotted together with ATP, ADP, THZ, HMP-P or HMP-PP as carriers onto PEI-cellulose F-coated Polygram sheets (MERCK, Germany). Ascending thin layer chromatography was performed in either 1 M formic acid containing 0.1 M LiCl or 1 M LiCl [7].

To analyse the reaction of *Sa*ThiE, advantage was taken of the reaction product of the previously performed *Sa*ThiM assay. Briefly, radioactive thiazole THZ-[^33^P], the product of the preceding *Sa*ThiM assay, was generated under standard conditions for 4 hours at 1 mM THZ and 500 µM [γ-^33^P]ATP and catalysis was terminated by heating. Subsequently either 400 µM HMP or HMP-P or HMP-PP was added to the formed THZ-[^33^P]. The reaction mixture was incubated at 37°C for an additional 30 min and stopped by heating at 95°C for 2 min. Subsequently 10 µl aliquots of the supernatants were spotted together with TMP and ATP as carriers onto PEI-cellulose F-coated Polygram sheets. Ascending thin layer chromatography was performed in 0.1 M formic acid containing 0.1 M LiCl.

*Sa*TPK kinetics were carried out according to [30] in 100 mM Tris-HCl buffer, pH 8.5, 4 mM MnCl_2_, 2 mM [8-^14^C]-ATP and 800 µM thiamine in a total volume of 100 µl. For analysis of substrate specificity, thiamine and TMP were used at a concentration of 400 µM. The reaction mixture was incubated at 37°C for 30 min and stopped by heating at 95°C for 2 min. Aliquots of 10 µl of the supernatants were spotted together with TPP, TMP and ATP as carrier onto PEI-cellulose F-coated Polygram sheets. The chromatograms were developed with ascending thin layer chromatography in 1 M LiCl.

The activity of *Sa*TenA was analysed in 100 µl 100 mM Tris-HCl, pH 7.5, containing either 1 mM thiamine or 1 mM TMP or 1 mM TPP and incubated for 30 min at 37°C. The reaction was stopped by heating at 95°C for 2 min and the reaction products were analysed by an assay where the amount of generated HMP-[^33^P] or THZ-[^33^P] was determined as described for the ThiD- or ThiM-assay, respectively.

Spots on the thin layer chromatograms of all experiments were identified by UV. The localization of radioactivity was visualised by exposure to X-ray films (Retina, Germany); spots were excised and transferred to vials containing scintillation fluid (Ultima Gold, Perkin Elmer, USA) and the counts per minute (CPM) obtained were quantified with the aid of the TRI-CARB 2000CA (United Instruments Packard, USA). The amount of the *de novo* synthesised HMP-[^33^P] and THZ-[^33^P] were calculated from the derived radioactive spots and the used substrate concentrations according to [7] and the results were analysed using GraphPad PRISM 4 (GraphPad software); the *K*_m_^app^-values were calculated from reciprocal Lineweaver-Burk plots.

The activity profile of the *Sa*GTPase was analysed in 96-well microtiter plate by performing a spectrophotometric assay with minor modifications [48], [49]. Briefly, the reaction was carried out at 37°C in a total volume of 100 µl 100 mM MOPS, pH 7.5, containing 1 mM MgCl_2_ and 1 mM of the respective substrates: GTP, ATP, CTP, ADP, AMP, TMP, TPP, glucose 6-phosphate, fructose 6-phosphate, ribose 5-phosphate, phosphoryl-ribose pyrophosphate and pyridoxal 5-phosphate. The reaction was stopped by addition of 50 µl of 25 mM ammonium molybdate in 4.5 M H_2_SO_4_. After 10 min incubation at room temperature 100 µl of 0.5 µM malachite green in 0.1% (w/v) poly(vinyl alcohol) was added and after 20 min the optical density (OD) of the reaction product at a wavelength of 620 nm was obtained using a Wavescan MCC 340 ELISA microplate reader (Labsystems, USA).

### TPP Binding Assay

The sequences of the −225 to −21 region (relative to the translation start-site) of the proposed operons consisting of *S. aureus gtpase*, *epi* and *tpk* as well as *tenA*, *thiM*, *thiD* and *thiE* were amplified by PCR (PCR-program: Denaturation for 5 min at 95°C, followed by 30 cycles of 45 s 95°C, 90 s at 48°C, 1 min at 68°C using *Pfu*-Polymerase, *S. aureus* ATCC 25923 genomic DNA and the sequence specific antisense and sense oligonucleotides as indicated in Table 2. The consensus sequence of the T7 promotor was introduced into the respective sense oligonucleotides. The PCR products were purified by PCR purification (Qiagen, Germany) and applied in RNA polymerase transcription using the MEGAscript *in vitro* transcription kit (Ambion, USA) according to the manufacture's recommendation. The resulting RNA was treated with RNAse-free DNAse (Ambion) at 37°C for 1 h, before it was phenol-chloroform extracted and suspended in 1x transcription buffer according to [50]. Subsequently the isolated RNA was denaturated at 65°C and gradiently cooled to 40°C according to [38]. Equimolar concentrations of RNA (MW of *gtpase*-*epi*-*tpk*-UTR: 66276 g/mol; MW of *tenA*-*thiM*-*thiD*-*thiE*-UTR: 66556 g/mol) and TPP were incubated at 37°C for 45 min and the mixture filtered through a Nanosep 10K Omega filter (Pall Corporation, USA). The concentration of unbound TPP in the flowthrough was determined by oxidation of TPP to thiochrome using 8.6 mM potassium ferricyanide in 1.4 M NaOH according to [38]. Thiochrome fluorescence was measured at an excitation wavelength of 365 nm and an emission wavelength at 455 nm using the fluorescent reader SFM25 (Kontron Instruments, USA). The obtained fluorescence was expressed as percentage of column bound TPP relative to the used amount of TPP.
